# Overexpression of miR-26b decreases the cisplatin-resistance in laryngeal cancer by targeting ATF2

**DOI:** 10.18632/oncotarget.20784

**Published:** 2017-09-08

**Authors:** Linli Tian, Jiarui Zhang, Xiuxia Ren, Xinyu Liu, Wei Gao, Chen Zhang, Yanan Sun, Ming Liu

**Affiliations:** ^1^ Department of Otorhinolaryngology, Head and Neck Surgery, The Second Affiliated Hospital, Harbin Medical University, Harbin, China, 150086

**Keywords:** laryngeal cancer, Hep-2/R, miR-26b, ATF2, cisplatin

## Abstract

Cisplatin is a common used chemotherapeutic drug for the treatment of laryngeal cancer. However, drug-resistance is a major obstacle in platinum-based chemotherapy for laryngeal cancer. Recent studies have demonstrated that dysregulation of microRNAs (miRNAs) is responsible for chemoresistance in multiple cancers including laryngeal cancer, but the potential mechanisms are required to be explored. In the present study, we constantly exposed the laryngeal cancer cell line Hep-2 with cisplatin to establish a cisplatin-resistant laryngeal cancer cell model (Hep-2/R). We found that Hep-2/R cells exhibited obvious resistance to cisplatin compared to the Hep-2 cells. However, overexpression of miR-26b significantly decreased the half maximal inhibitory concentration (IC50) of cisplatin to Hep-2/R. Mechanically, miR-26b in Hep-2/R decreased the expression of ATF2, and thus inhibiting the phosphorylation of ATF2 and formation of cellular ATF2-c-Jun complex induced by cisplatin. As the results, Hep-2/R cells failed to overexpress the Bcl-xl which is a key anti-apoptotic protein under the cisplatin treatment. Therefore, overexpression of miR-26b was found to be able to promote mitochondrial apoptosis induced by cisplatin.

## INTRODUCTION

Laryngeal cancer occupies the first position among the head and neck malignant tumors. Generally, surgery is the most effective treatment approach for early-stage laryngeal cancer. However, a large proportion of laryngeal cancer patients have suffered from the advanced-stage disease at the first diagnosis [1, 2]. Because of the wide metastasis of cancer cells, surgery is not sufficient to extirpate the tumor completely for these patients with advanced-stage laryngeal cancer, but chemotherapy is a feasible strategy to improve the patient’s prognosis [3, 4]. However, chemoresistance is a common phenomenon when the cancer cells were constantly exposed to chemotherapeutic drugs f in laryngeal cancer patients [5].

Cisplatin is a common used chemotherapeutic drug for the treatment of cancers including laryngeal cancer [6, 7]. It acts as the cytotoxic agent in cancer cells by damaging DNA and inducing apoptosis. Mechanically, cisplatin in cancer cells binds to nitrogen atoms of DNA base, and thus forming the cross-link with cellular DNA. DNA damage may trigger DNA repair processes, and the failure of which may trigger apoptosis in cancer cells [8-10]. Although cisplatin treatment may achieve satisfactory effects at the beginning, long-term exposure to cisplatin may induce acquired drug resistance in laryngeal cancer [6, 11, 12].

MicroRNAs (miRNAs) are endogenous and non-coding RNAs with single chain of 18#x007E;25 nucleotides in length [13, 14]. Generally, miRNAs suppress gene expression by binding to their target mRNA at the 3′-untranslated region (3′ UTR), and thus they are considered as the important negative regulators of gene expression in cancer cells. It has been demonstrated that dysregulation of miRNAs is required in tumorigenesis and tumor development [15-17]. Furthermore, Aberrant expression of miRNAs is found to induce chemoresistance in various cancers including laryngeal cancer [18-20]. The present study demonstrates that reduction of miR-26b is associated with chemoresistance in cisplatin-resistant laryngeal cancer cell model, and recovery of miR-26b is able to decrease this cisplatin resistance by targeting ATF2.

## RESULTS

### Recovery of miR-26b weakens cisplatin resistance in Hep-2/R

**Figure 1:**
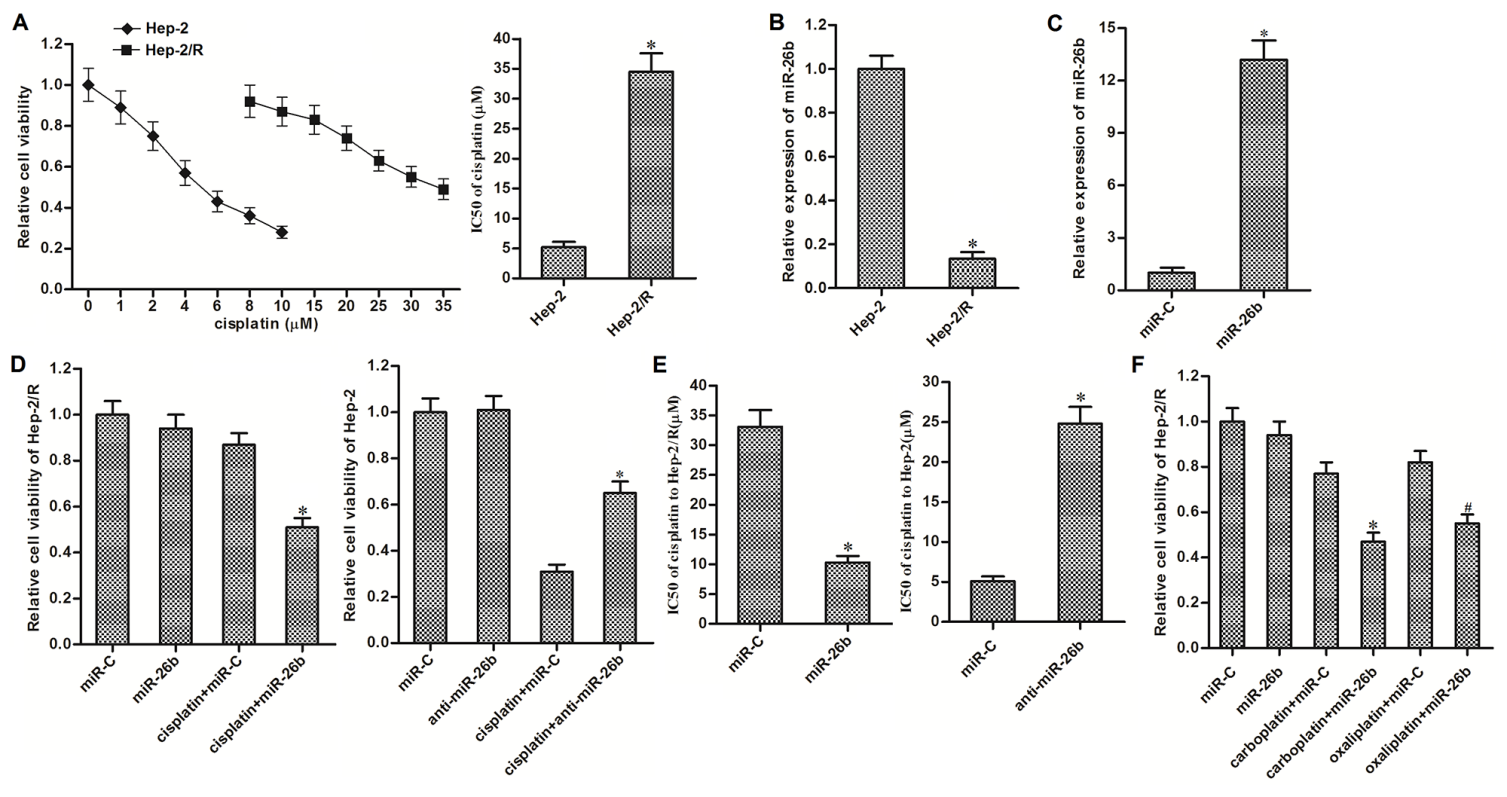
Recovery of miR-26b weakened cisplatin resistance in Hep-2/R **(A)** MTT assays were performed to evaluate the sensitivity of Hep-2 and Hep-2/R cells to cisplatin. *P<0.05 *vs.* Hep-2 group. **(B)** QRT-PCR analysis was performed to detect the expression of miR-26b in Hep-2 and Hep-2/R. *P<0.05 *vs.* Hep-2 group. **(C)** 50 pmol/ml miR-C or miR-26b mimics was transfected into Hep-2/R cells. Cellular level of miR-26b was detected by qRT-PCR analysis. *P<0.05 *vs.* miR-C group. **(D)** Hep-2/R and Hep-2 cells were transfected with miR-26b and anti-miR-26b (50 pmol/ml) respectively before treatment with 10 μM cisplatin for 48 h. Cell viability of was determined by MTT assays. *P<0.05 *vs.* cisplatin+miR-C group. **(E)** Effect of miR-26b and anti-miR-26b on chaning the IC50 of cisplatin to Hep-2/R and Hep-2. *P<0.05 *vs.* miR-C group. **(F)** Hep-2/R cells were transfected with miR-26b or miR-C (50 pmol/ml) before treatment with 10 μM carboplatin or oxaliplatin for 48 h. Cell viability of Hep-2/R was determined by MTT assays. *P<0.05 *vs.* carboplatin+miR-C group. ^#^*P*<0.05 *vs.* oxaliplatin+miR-C group.

To study the acquired cisplatin resistance in laryngeal cancer, we established the cisplatin-resistant Hep-2 cells (Hep-2/R) by constantly exposure with cisplatin. Results of MTT assays showed that sensitivity of Hep-2/R cells was significantly lower than their corresponding Hep-2 cells. Half maximal inhibitory concentration (IC50) of cisplatin to Hep-2/R was increased about 6 fold compared to the Hep-2 cells (Figure 1A). On the other hand, we found the significant difference of miR-26b expression between Hep-2/R and Hep-2. Expression of miR-26b was significantly decreased in Hep-2/R cells compared to the Hep-2 cells (Figure 1B). We therefore investigated the association between miR-26b and cisplatin resistance in Hep-2/R. As transfection with miR-26b mimics significantly recovered the cellular level of miR-26b in Hep-2/R (Figure 1C), we subsequently performed MTT assays to evaluate the effect of miR-26b on cisplatin sensitivity in Hep-2/R. In addition, as 10 μM cisplatin only induced slight cell death of Hep-2/R, we chose this concentration of cisplatin for co-treatment with miR-26b in the following experiments. As shown in Figure 1D, recovery of miR-26b significantly enhanced the effect of cisplatin on killing Hep-2/R, whereas inhibition of miR-26b decreased the sensitivity of routine Hep-2 cells to cisplatin. Specifically, transfection with miR-26b decreased the IC50 of cisplatin to Hep-2/R by about 68% compared to the control miRNA (miR-C) group. On the other hand, knockdown of miR-26b increased the IC50 of cisplatin to Hep-2/R by about 3.77 fold compared to the control group (Figure 1E). In addition, we next tested whether miR-26b reduced the cross-resistance of Hep-2/R to carboplatin and oxaliplatin. As shown in Figure 1F, transfection with miR-26b was able to sensitize Hep-2/R cells to either carboplatin or oxaliplatin. These data suggested that aberrant expression of miR-26b may be responsible for acquired resistance to platinum-based chemotherapeutic agents in laryngeal cancer, which can be weakened by recovery of miR-26b.

### miR-26b targets ATF2 in Hep-2 and Hep-2/R

**Figure 2:**
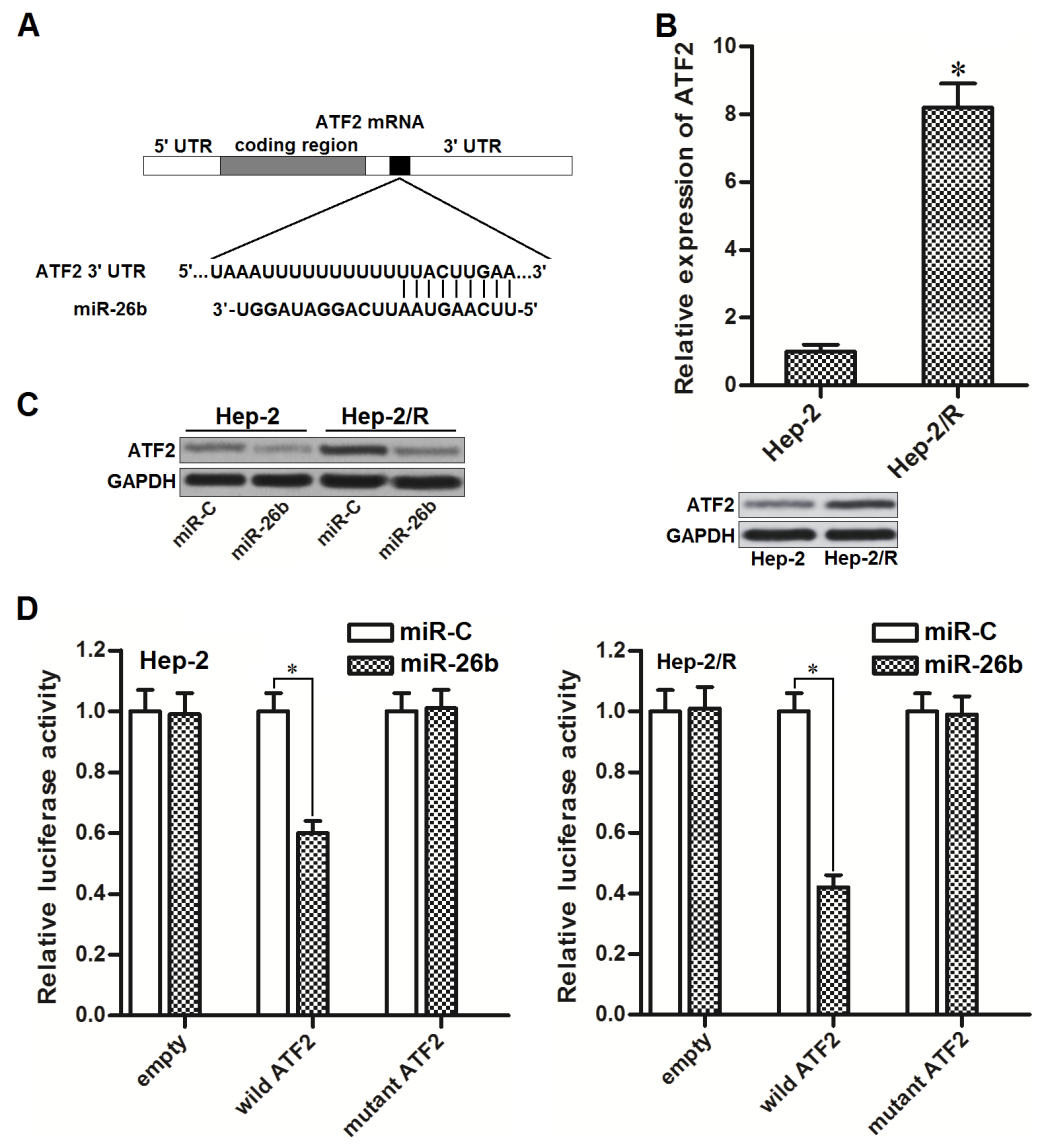
miR-26b targets ATF2 in Hep-2 and Hep-2/R **(A)** ATF2 mRNA 3’ UTR contained predicted binding sequence paired with miR-26b. **(B)** QRT-PCR and western blot analysis was performed to detect the expression of ATF2 at mRNA level and protein level in Hep-2 and Hep-2/R cells. *P<0.05 *vs.* Hep-2 group. **(C)** Effect of miR-26b on decreasing the expression of ATF2 in Hep-2 and Hep-2/R cells. **(D)** Relative luciferase activities in Hep-2 and Hep-R cells were measured by using the Dual-Luciferase Reporter System. *P<0.05.

To search the potential target of miR-26b, public databases of TargetScan (www.targetscan.org), miRanda (http://www.microrna.org) and PicTar (http://pictar.mdc-berlin.de) were used. We observed that mRNA 3’ UTR of activating transcription factor 2 (ATF2) contained putative binding sequences paired with miR-26b (Figure 2A). Furthermore, ATF2 in Hep-2/R was overexpressed compared to the Hep-2 cells (Figure 2B). It suggested that ATF2 is the target of miR-26b in Hep-2 and Hep-2/R. To test whether miR-26b regulated ATF2 expression in Hep-2 and Hep-2/R, these cells were transfected with miR-26b mimics before detecting the expression of ATF2 by western blot analysis. As shown in Figure 2C, protein level of ATF2 was obviously decreased in both Hep-2 and Hep-2/R cells after they were transfected with miR-26b. Moreover, results of luciferase reporter assay showed that co-transfection with miR-26b decreased the luciferase activity of reporter contained wild but not the mutant ATF2 3’UTR (Figure 2D). Token together, these results demonstrated that miR-26b targeted ATF2 in Hep-2 and Hep-2/R cells.

### Overexpression of ATF2 is responsible for cisplatin resistance in Hep-2/R

**Figure 3:**
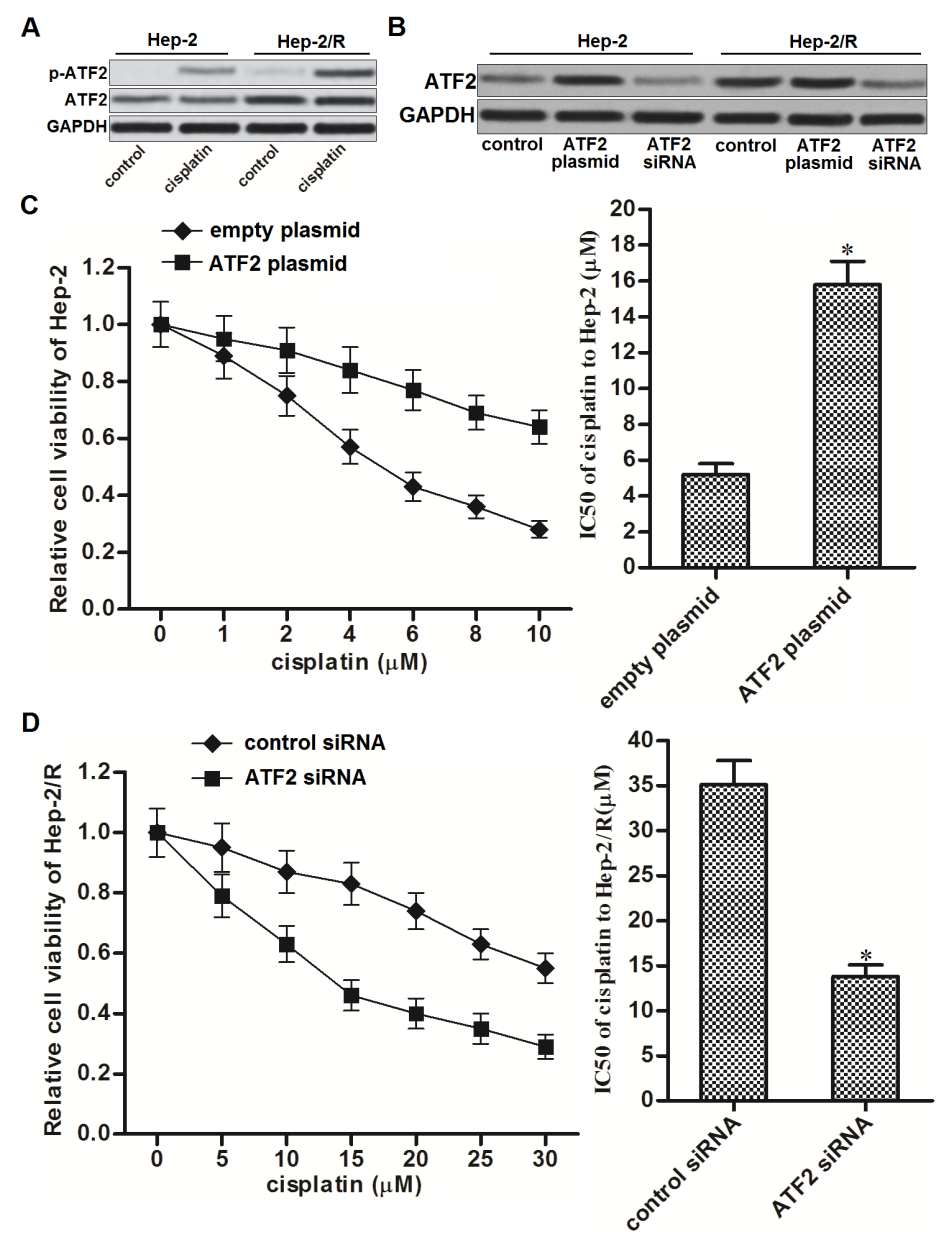
Overexpression of ATF2 is responsible for cisplatin resistance in Hep-2/R **(A)** Phosphorylation level of ATF2 in Hep-2 and Hep-2/R cells after they were treated with cisplatin (10 μM). **(B)** Effect of ATF2 plasmid and siRNA on changing the cellular level of ATF2 in Hep-2 and Hep-2/R cells. **(C)** Transfection with ATF2 plasmid increased the IC50 of cisplatin to Hep-2 *P<0.05 *vs.* empty plasmid group. **(D)** Transfection with ATF2 siRNA decreased the IC50 of cisplatin to Hep-2/R. *P<0.05 *vs.* control siRNA group.

Cisplatin treatment induced phosphorylation of ATF2 in cancer cells. Since the expression of ATF2 in Hep-2/R was significantly higher than that in Hep-2, phosphorylation level of ATF2 in Hep-2/R was more obvious than the Hep-2 cells after they were treated with equal concentration of cisplatin (Figure 3A). To investigate whether overexpression of ATF2 was responsible for cisplatin resistance in Hep-2/R, ATF2 eukaryotic expression plasmid and siRNA was introduced into the Hep-2 and Hep-2/R cells to change the cellular level of ATF2. As shown in Figure 3B, transfection with ATF2 plasmid increased the protein level of ATF2 in Hep-2 and Hep-2/R, whereas the ATF2 siRNA decreased the protein level of ATF2 in them. We found that introduction with ATF2 plasmid in Hep-2 decreased its sensitivity to cisplatin (Figure 3C). On the contrary, knockdown of ATF2 in Hep-2/R significantly decreased the IC50 of cisplatin to it (Figure 3D). These data indicated that cellular level of ATF2 was associated with sensitivity to cisplatin, and overexpression of ATF2 was responsible for cisplatin resistance in Hep-2/R.

### miR-26b reverses cisplatin resistance of Hep-2/R through inhibiting the expression of ATF2

**Figure 4:**
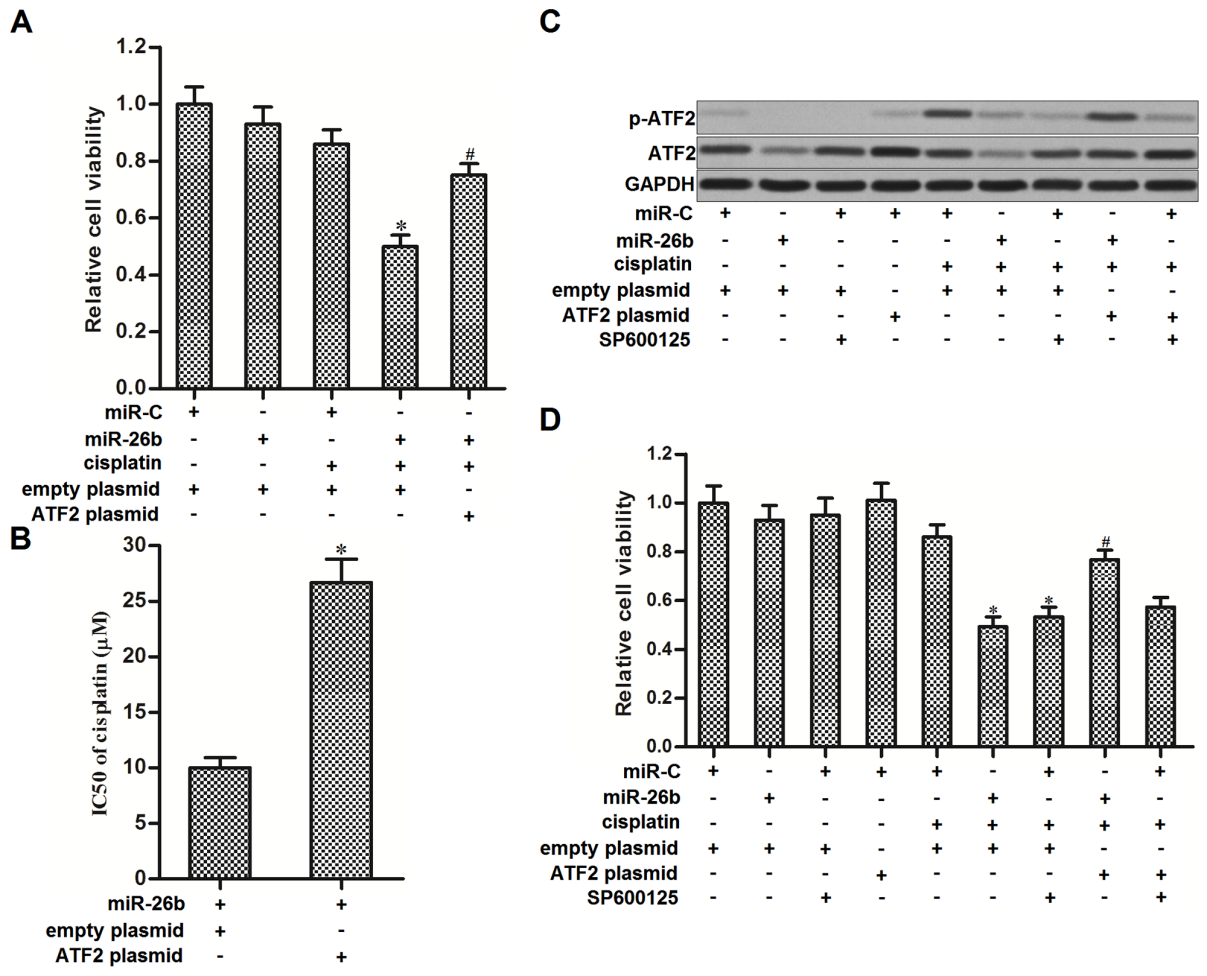
Overexpression of miR-26b sensitized Hep-2/R to cisplatin through inhibiting the phosphorylation of ATF2 **(A)** Hep-2/R cells were transfected with miR-26b and ATF2 plasmid before treated with cisplatin (10 μM). Cell viability was then measured by using MTT assays. *P<0.05 *vs.* cisplatin+miR-C+empty plasmid group. ^#^*P*<0.05 *vs.* cisplatin+miR-26b+empty plasmid group. **(B)** Transfection with ATF2 plasmid increased the IC50 of cisplatin to Hep-2/R cells transfected with miR-26b. *P<0.05 *vs.* miR-26b+empty plasmid group. **(C)** Effect of cisplatin (10 μM), miR-26b, SP600125 (50 μM) and ATF2 plasmid on influencing the phosphorylation of ATF2 in Hep-2/R. **(D)** Effect of cisplatin (10 μM), miR-26b, SP600125 (50 μM) and ATF2 plasmid on influencing the cell viability of Hep-2/R. *P<0.05 *vs.* cisplatin+miR-C+empty plasmid group. ^#^*P*<0.05 *vs.* cisplatin+miR-26b+empty plasmid group.

We next performed experiments to explore whether miR-26b reverses cisplatin resistance of Hep-2/R by targeting ATF2. As the gene of ATF2 on recombinant plasmid was lack of 3’ UTR, we transfected the Hep-2/R cells with ATF2 plasmid against the effect of miR-26b on inhibiting ATF2. As shown in Figure 4A, transfection with ATF2 plasmid significantly weakened the effect of miR-26b on promoting cisplatin-induced cell death in Hep-2/R, and the effect of miR-26b on reducing the cisplatin IC50 to Hep-2/R was declined due to the ATF2 plasmid transfection (Figure 4B). These data indicated that miR-26b reversed cisplatin resistance of Hep-2/R by targeting ATF2. Phosphorylation of ATF2 is regulated by c-Jun N-terminal kinase (JNK) signaling [21]. We therefore treated the Hep-2/R cells with SP600125 which is the JNK specific inhibitor [22] to impede the phosphorylation of ATF2. We observed that cisplatin treatment induced significant phosphorylation of ATF2 in Hep-2/R. However, both miR-26b and SP600125 abolished cisplatin-induced phosphorylation of ATF2 in Hep-2/R. In addition, transfection with ATF2 plasmid recovered the phosphorylation of ATF2 in cisplatin and miR-26b co-treated Hep-2/R cells. However, ATF2 plasmid failed to recover the phosphorylation of ATF2 in cisplatin and SP600125 co-treated Hep-2/R cells, because ATF2 is the downstream of JNK signaling (Figure 4C). The results of MTT assays showed that SP600125 promoted cisplatin-induced cell death in Hep-2/R, similarly as the miR-26b. In addition, enforced expression of ATF2 inhibited the synergistic effect of miR-26b on cisplatin-induced cell death. However, ATF2 plasmid can not rescue the Hep-2/R cells co-treated with SP600125 and cisplatin, because SP600125 inhibited phosphorylation of ATF2 even the ATF2 was overexpressed (Figure 4D). Taken together, these data indicated that miR-26b decreased the expression of ATF2, subsequently suppressed the phosphorylation of ATF2 induced by cisplatin, and thus reversing the cisplatin resistance to Hep-2/R.

### miR-26b promoted mitochondrial apoptosis in cisplatin-treated Hep-2/R

**Figure 5:**
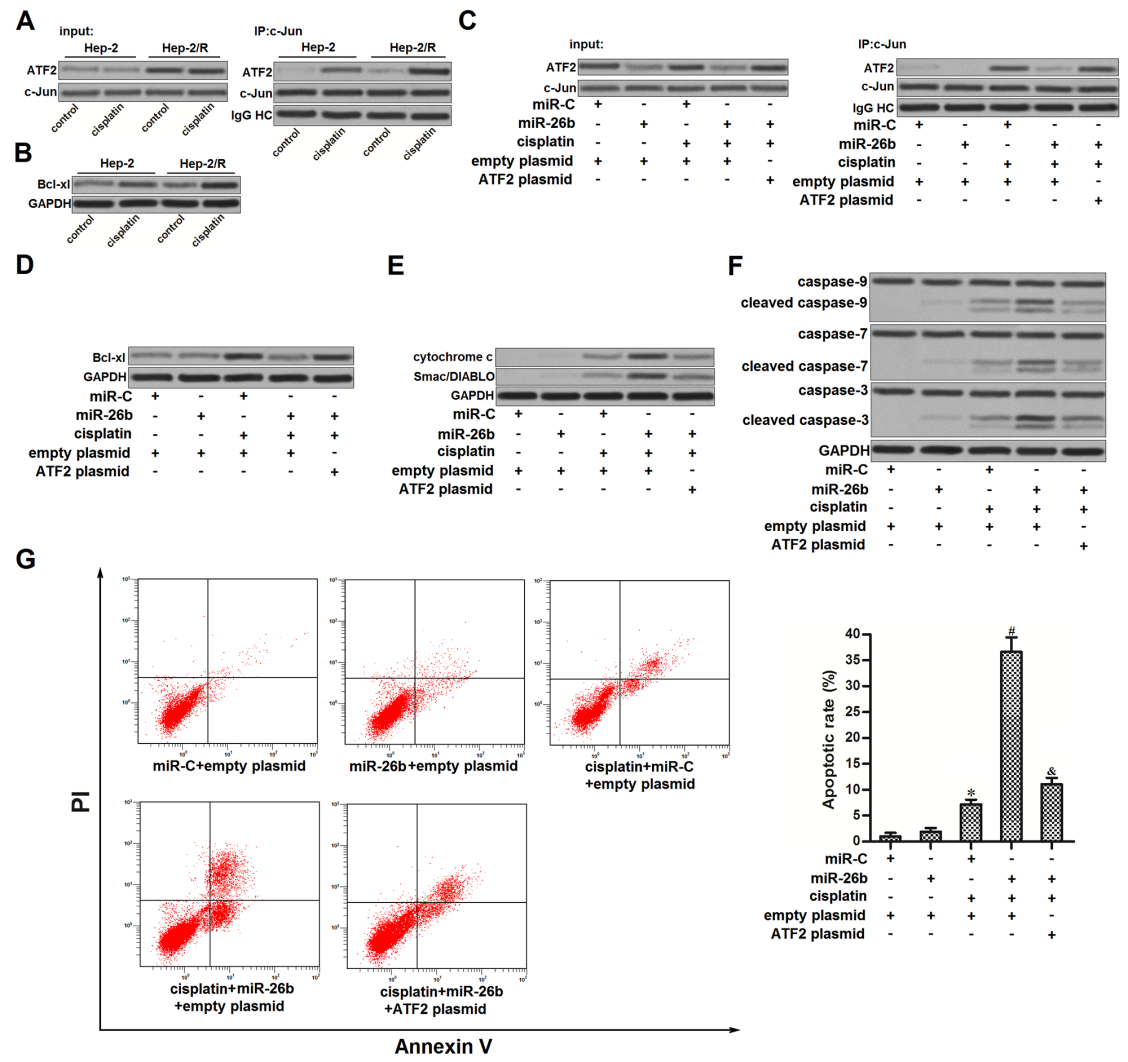
miR-26b promoted mitochondrial apoptosis in cisplatin-treated Hep-2/R **(A)** Co-immunoprecipitation was performed to evaluate the interaction with ATF2 and c-Jun in Hep-2 and Hep-2/R cells after they were treated with cisplatin (10 μM). **(B)** Western blot analysis was performed to detect the expression of Bcl-xl in Hep-2 Hep-2/R cells after they were treated with cisplatin (10 μM). **(C)** Hep-2/R cells were transfected with miR-26b and ATF2 plasmid before treatment with cisplatin (10 μM). Co-immunoprecipitation was performed to evaluate the interaction with ATF2 and c-Jun. **(D)** Hep-2/R cells were transfected with miR-26b and ATF2 plasmid before treatment with cisplatin (10 μM). Western blot analysis was performed to detect the expression of Bcl-xl. **(E)** Mitochondria in Hep-2/R were removed before detecting the cellular level of cytochrome c and Smac/DIABLO in cytoplasm. **(F)** Activation of caspase-9, -7, and -3 in Hep-2/R was evaluated by western blto analysis. **(G)** Cell apoptosis of Hep-2/R was detected by flow cytometry. *P<0.05 *vs.* miR-C+empty plasmid group. ^#^*P*<0.05 *vs.* cisplatin+miR-C+empty plasmid group. ^&^*P*<0.05 *vs.* cisplatin+miR-26b+empty plasmid group.

ATF2 is a common co-activator with c-Jun [23]. As cisplatin induced significantly higher level of phosphorylated ATF2 in Hep-2/R compared to the Hep-2 cells (Figure 3A), we observed more significant interaction with ATF2 and c-Jun in Hep-2/R cells treated with cisplatin (Figure 5A). Subsequently, we observed that cisplatin induced obviously higher level of B-cell lymphoma-extra large (Bcl-xl) in Hep-2/R compared to the Hep-2 cells (Figure 5B). It may because heterodimers of ATF2 and c-Jun promote the expression of Bcl-xl which is an anti-apoptotic protein [24]. However, we found that recovery of miR-26b in Hep-2/R inhibited the interaction with ATF2 and c-Jun (Figure 5C). As the downstream, miR-26b inhibited the expression of Bcl-xl in cisplatin-treated Hep-2/R (Figure 5D). Bcl-xl is an pro-survival against mitochondrial apoptosis [25]. In Hep-2/R cells, we observed that recovery of miR-26b obviously promoted the release of cytochrome c and Smac/DIABLO which were the mitochondria-derived apoptotic inducers into the cytoplasm (Figure 5E). As the results, combination with cisplatin and miR-26b induced significant activation of caspase-9, -7 and -3 (Figure 5F), followed by apoptosis occurrence (Figure 5G). These results demonstrated that recovery of miR-26b was able to reverse the apoptotic resistance in cisplatin-treated Hep-2/R.

### miR-26b reverses cisplatin resistance of Hep-2/R in vivo

**Figure 6:**
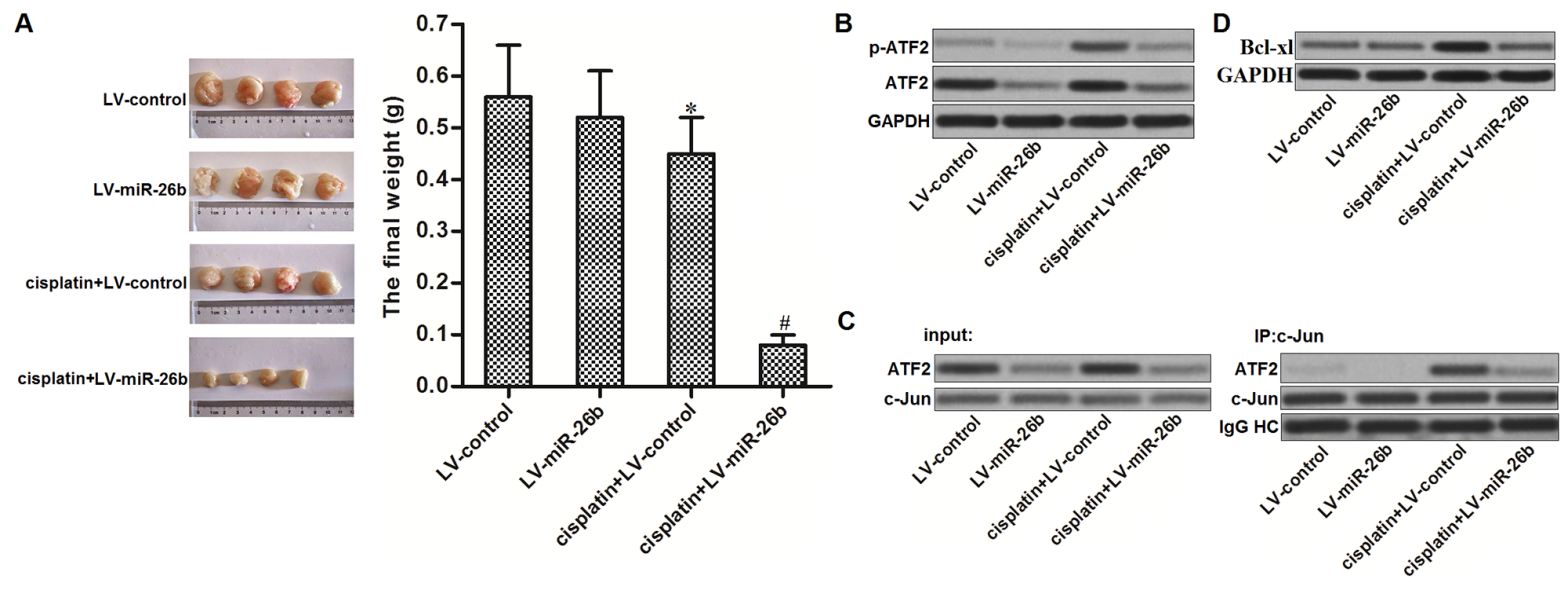
Overexpression of miR-26b reverses cisplatin resistance of Hep-2/R *in vivo* **(A)** Mice were inoculated with miR-26b-overexpressed or control Hep-2/R cells. Subsequently, they were received cisplatin treatment with equal dose (2 mg/kg). The final xenografts were separated and weighted. *P<0.05 *vs.* LV-control group. ^#^*P*<0.05 *vs.* Cisplatin+LV-control group. **(B)** Cells from xenografts were purified by using collagenase type III. Phosphorylation of ATF2 in these cells was evaluated by western blot analsyis. **(C)** Interaction with ATF2 and c-Jun in xenograft was evaluated by co-immunoprecipitation assays. **(D)** Expression of Bcl-xl in xenograft was evaluated by western blot analsyis.

For *in vivo* experiments, miR-26b-overexpressed or control Hep-2/R cells were inoculated into mice. We found that Hep-2/R-formed tumors were resistant to cisplatin treatment *in vivo*. However, the miR-26b-overexpressed xenografts were obviously more sensitive to cisplatin treatment compared to the control xenografts (Figure 6A). In the removed tumor tissues, cisplatin treatment induced obvious phosphorylation of ATF2 in control Hep-2/R cells. However, phosphorylation of ATF2 in miR-26b-overexpressed Hep-2/R was still slight when the mice were underwent cisplatin treatment (Figure 6B). As the downstream of ATF2 pathway, we observed that overexpression of miR-26b obviously inhibited the cisplatin-dependent interaction with ATF2 and c-Jun in the xenografts (Figure 6C). As the results, expression level of Bcl-xl in miR-26b-overexpressed Hep-2/R was obviously lower than the control Hep-2/R when the mice were underwent cisplatin treatment (Figure 6D). Token together, we demonstrated that miR-26b reversed cisplatin resistance of Hep-2/R through decreasing the expression of ATF2 *in vivo*.

## DISCUSSION

miR-26b has been reported to act as a negative regulator for tumorigenesis and tumor proliferation, invasion and migration [26-28]. Therefore, reduction of miR-26b expression correlates with poor clinical outcome of cancer patients [29, 30]. Furthermore, recent studies demonstrate that reduction of miR-26b is responsible for chemoresistance in some cancers such as nasopharyngeal carcinoma, hepatocellular carcinoma and lung cancer. Overexpression of miR-26b sensitizes these cancer cells to cisplatin and doxorubicin [31-33]. These previous reports suggest that miR-26b is a suppressor for chemoresistance. However, the potential mechanisms is still needed to be explored in cancers, especially in laryngeal cancer.

Cisplatin is a common used chemotherapeutic drug for the treatment of laryngeal cancer. However, acquired resistance is a major limitation in platinum-based chemotherapy for laryngeal cancer [6, 11, 12]. Agreement with the previous reports, our data demonstrated that reduction of miR-26b expression participated in the induction of acquired cisplatin resistance in laryngeal cancer. However, we found that recovery of miR-26b re-sensitized the cisplatin-resistant laryngeal cancer to cisplatin treatment both *in vitro* and *in vivo*. Therefore, we declare that miR-26b is a suppressor for cisplatin resistance in laryngeal cancer.

Activating transcription factor 2 (ATF2) belongs to cAMP response element binding family. Activation of JNK phosphorylates threonine residues of ATF2 to trigger it [34, 35]. Studies have reported that damage of DNA induced phosphorylation of ATF2 through the JNK pathway. Activated ATF2 then targets several pro-survival molecules to promote DNA repair and cell survival. Therefore, phosphorylation of ATF2 induces resistance to DNA-damaging agents such as cisplatin [36-38].

In this study, we found that ATF2 was overexpressed in cisplatin-resistant laryngeal cancer cells. Therefore, cisplatin treatment induced higher level of phosphorylated ATF2 in these resistant laryngeal cancer cells. We then proved that overexpression of ATF2 was responsible for cisplatin resistance in laryngeal cancer. Furthermore, we demonstrated that miR-26b decreased the expression of ATF2 to inhibit cisplatin-induced phosphorylation of ATF2. Thus, miR-26b overexpression was proved to be able reverse the acquired cisplatin resistance laryngeal cancer. In addition, SP600125, a JNK specific inhibitor [22], also decreased the cisplatin resistance, because SP600125 inhibited the phosphorylation of ATF2 through the JNK pathway. These evidence demonstrated the importance of miR-26b in inhibiting cisplatin resistance of laryngeal cancer.

Cisplatin-induced damage of DNA induces mitochondrial apoptosis, which can be inhibited by overexpression of anti-apoptotic family proteins such as Bcl-xl. Phosphorylated ATF2 can interact with c-Jun which is a nuclear transcription factor to promote the expression of pro-survival protein of Bcl-xl, and thus inhibiting mitochondrial apoptosis [25, 39, 40]. We found that recovery of miR-26b in cisplatin-resistant laryngeal cancer inhibited interaction with ATF2 and c-Jun by decreasing the cellular level of ATF2. Subsequently, miR-26b abolished cisplatin-induced overexpression of Bcl-xl and thus promoting the mitochondrial apoptosis in these cisplatin-resistant laryngeal cancer cells underwent cisplatin treatment.

In summary, our study has provided several evidence that overexpression of miR-26b decreases the cisplatin-resistance in laryngeal cancer by targeting ATF2. Further studies are required to evaluate the novel strategy of adjuvant treatment with miR-26b for reversing resistance in platinum-based chemotherapy in laryngeal cancer.

## MATERIALS AND METHODS

### Cell culture

Human laryngeal cancer cell line Hep-2 was obtained from the Institute of Biochemistry and Cell Biology, Shanghai Institute for Biological Sciences, Chinese Academy of Sciences (Shanghai, China). Cisplatin-resistant Hep-2 cell model (Hep-2/R) was established by constant exposure to cisplatin. Hep-2 cells were cultured in RPMI-1640 medium containing 10% fetal bovine serum (FBS, Gibco, USA) at 37 °C with 5% CO_2_. Hep-2/R cells were cultured in RPMI-1640 medium supplemented with 10% FBS and 2 μM cisplatin at 37 °C with 5% CO_2_. To eliminate the interference of residual cisplatin in Hep-2/R culture system, Hep-2/R cells were moved to the cispatin-free medium for 2 weeks before following experiments.

### RNA reversed transcription and quantitative real-time PCR (qRT-PCR) analysis

Total RNA of Hep-2 and Hep-2/R cells were extracted by using Trizol reagent (Invitrogen, USA). Reverse transcription (20 ng/μl of total RNA) of miR-26b and ATF2 was performed by using PrimeScript RT reagent Kit (TaKaRa, Japan) with stem-loop RT primer (5’-CTCAACTGGTGTCGTGG AGTCGGCAATTCAGTTGAGACCTATCC-3’) and paired primer (ATF2 forward: 5’-TACAAGTGGTCGTCGG-3’, reverse: 5’-CGGTTACAGGGCAATC-3’), respectively. Real-time PCR analysis was carried-out on an ABI PRISM 7900 Sequence Detection System (Applied Biosystems, USA) by using SYBR Premix Ex Taq (TaKaRa). U6 snRNA and GAPDH were chosen as the endogenous control for detection of miR-26b and ATF2, respectively.

### Transfection

Mature human miR-26b (5’-UUCAAGUAAUUCAGGAUAGGU-3’), miR-26b inhibitor (anti-miR-26b, 5’-ACCUAUCCUGAAUUACUUGAA-3’) and control microRNA sequence (miR-C, 5’-GUUCUAGUACAAUAUUAGGAG-3’) were purchased from RiboBio Co. Ltd (Guangzhou, China). For overexpression of ATF2, open reading frame region of human ATF2 was amplified and linked to pcDNA3.1 eukaryotic expression plasmid (Invitrogen, USA). For knockdown of ATF2, ATF2 siRNA was purchased from Genepharma Company (Shanghai, China). For transfection, 2 μg/ml ATF2 plasmid, 50 pmol/ml miR-26b, anti-miR-26b, miR-C and ATF2 siRNA were introduced into the Hep-2 and Hep-2/R cells by using lipofectamine 2000 (Invitrogen) according to the manufacturer’s instructions.

### Drug sensitivity assay

Transfected Hep-2 and Hep-2/R cells were seeded into 96-well plates overnight before treatment with various concentrations of cisplatin for 48 h. Subsequently, MTT assays were performed to measure the cell viability of them. Half maximal inhibitory concentration (IC50) of cisplatin to Hep-2 and Hep-2/R was determined according to the cell viability curves.

### Luciferase reporter assay

3′ UTR of human ATF2 gene was cloned into the pGL3 luciferase reporter plasmid (Promega, USA) downstream of luciferase gene. QuikChange Site-Directed Mutagenesis kit (Stratagene, USA) was used to mutate the sequence of ATF2 3’ UTR which was the complementary binding site of miR-26b in recombinant luciferase reporter plasmid. Hep-2 and Hep-2/R cells were seeded into 48-well plates overnight before co-transfection with luciferase reporter plasmids, pRL-TK renilla plasmids (Promega) and miR-26b by using lipofectamine 2000. 24 h later, luciferase reporter activities were measured by using a Dual Luciferase Reporter Assay Kit (Promega).

### Mitochondria removal

Mitochondria/Cytosol Fraction Kit (BioVision, USA) was used to remove the mitochondria in treated Hep-2/R cells. Subsequently, cytoplasm fraction free of mitochondria was collected for following western blot analysis of cytochrome c.

### Immunoprecipitation

Treated Hep-2 and Hep-2/R cells were lysed with RIPA buffer (Cell Signaling Technologies, USA) on ice for 30 min. Supernatant of cell lysate were incubated with primary antibody of c-Jun (Santa Cruze, USA) at 4 °C overnight. Subsequently, the supernatant were mixed with protein G agarose beads for 2 h at 4°C. Immunoprecipitates were washed three times with RIPA buffer for the following western blot analysis.

### Western blot analysis

Total proteins in Hep-2 and Hep-2/R cells were extracted by using RIPA lysis buffer on ice for 30 min. 50 μg total proteins were separated with SDS-PAGE before transferring to a nitrocellulose membrane. The membrane was then blocked with skimmed milk and incubated with primary antibody of ATF2, p-ATF2, c-Jun, Bcl-xl, cytochrome c, Smac/DIABLO, caspase-9, caspase-7, caspase-3 and GAPDH (dilution 1:200, Santa Cruze, USA) at 4 °C overnight. Subsequently, the membrane were labeled with HRP-conjugated secondary antibodies (dilution 1:5000, Santa Cruze, USA) for 2 h at room temperature. Proteins on the membrane were probed by using an enhanced chemiluminescent substrate (Thermo Fisher Scientific, Inc, USA).

### Cell apoptosis detection

Treated Hep-2/R cells were collected and washed three time. Apoptotic rate of these Hep-2/R cells was measured on a flow cytometry (Becton Dickinson, USA) by using an Annexin V-FITC apoptosis detection kit (Sigma Aldrich, USA).

### Xenograft tumor model

Recombinant lentivirus contained miR-26b precusor sequence (LV-miR-26b) were purchased from the Shanghai Genechem Co., Ltd. (Shanghai, China). Hep-2/R cells were transfected with LV-miR-26b or control lentivirus (LV-control) for preparing to inoculation on mice. Four-week-old female BALB/c nude mice (Shanghai Super-B&K Laboratory Animal Corp., Ltd, Shanghai, China) were subcutaneously injected with 5×10^6^ prepared Hep-2/R cells. Mice were treated with cisplatin i.p. twice a week (2 mg/kg) before sacrifice on day 28 post-injection. Tumor tissues were then resected and weighted. Cells from the tumor tissues were digested by using collagenase type III to detect the expression of ATF2 and Bcl-xl in these cells. Our experimental protocols and animal care were approved by the Animal Care Committee of The Second Affiliated Hospital, Harbin Medical University.

### Statistical analysis

Experiments were repeated at least 3 times and the experimental data were represented as mean ± SD. Differences between groups were analyzed by using SPSS 15.0 with Two-tail Student’s t test and ANOVA methods. *P*<0.05 was considered to be statistically significant.
